# The clinical application progress and potential of drug-induced sleep endoscopy in obstructive sleep apnea

**DOI:** 10.1080/07853890.2022.2134586

**Published:** 2022-10-21

**Authors:** Alonço Viana, Débora Estevão, Chen Zhao

**Affiliations:** a Graduate Program of Neurology, Rio de Janeiro State Federal University (UNIRIO), Rio de Janeiro, Brazil; b Department of Otorhinolaryngology, Marcilio Dias Naval Hospital, Rio de Janeiro, Brazil; c Department of Otorhinolaryngology, Glória D’Or Hospital - Rede D’Or São Luiz, Rio de Janeiro, Brazil; d Department of Otorhinolaryngology, the First Hospital of China Medical University, Shenyang, China

**Keywords:** Sleep endoscopy, drug-induced sleep endoscopy, obstructive sleep apnoea, upper airway, collapse patterns

## Abstract

**Background and Objective:** Obstructive sleep apnoea (OSA) is characterized by nocturnal repetitive upper airway (UA) collapse. For sleep physicians, the recognition of UA collapse characteristics is critical for understanding OSA mechanisms and developing individualized treatment plans. Drug-induced sleep endoscopy (DISE) is an exam during simulated sleep that allows the dynamic assessment of the UA of individuals with OSA. The initial recognition of DISE was to locate the sites of UA obstruction and direct the surgical selection of OSA since it was introduced in the 1990s. After approximately 30 years of studies, based on advances in endoscopic operative techniques and innovative treatments of OSA, DISE had been performed to explore mechanisms and comprehensive treatments related to UA collapse.

**Methods: **This article reviewed contemporary DISE advances, including indications and contraindications, technique of induced sleep, endoscopic operation, UA characteristics classification.

**Results and Conclusions:** Precise selection based on the association between collapse patterns and treatment modalities, such as continuous positive airway pressure, oral appliance, positional therapy, robotic surgery and neurostimulator implanting, is the future research prospect based on DISE.

**Key messages**
- DISE provides sleep physicians with valuable information about the upper airway collapse characteristics and dynamic changes during sleep.
- The studies based on DISE findings improve the selectivity and efficiency of treatment modalities, including classical therapies such as continuous positive airway pressure, oral appliance, positional therapy, and innovative therapies such as neurostimulator implanting and robotic surgery, promote the advancement of OSA precision medicine

## 1. Introduction

Obstructive sleep apnoea (OSA) is characterized by the recurrence of respiratory disorder events associated with decreased oxygen saturation and awakenings during sleep, causing cardiovascular, occupational, and neurocognitive consequences and increasing morbidity and mortality [1]. In 1999, the American Academy of Sleep Medicine (AASM) defined criteria for determining disease diagnosis and severity using the apnoea-hypopnoea index (AHI) obtained on polysomnography (PSG). From this index, the prevalence in the adult population is quite variable: 9 to 38% for AHI ≥5 events/h, and 6 to 17% for AHI ≥15 events/h. Several phenotypic characteristics, such as male sex, obesity, increased age, cervical circumference and increased waist circumference, craniofacial deformities, and race, contribute to OSA. The treatments include continuous positive airway pressure (CPAP), oral appliance (OA), positional therapy, and surgery [2,3].

The requirement for precisely evaluating UA collapse in individuals with OSA and improving surgical results promotes the appearance of drug-induced sleep endoscopy (DISE) in the 1990s. DISE is an exam that enables the dynamic study of the upper airway (UA) during sleep induced by drugs. Observation through DISE can find the anatomical deformity and muscular dysfunction with partial or complete collapse, involvement of one or more sites, and different configurations of UA collapse during episodes of apnoea and hypopnoea and can guide the choice of treatment based on various findings. After nearly two decades of research, DISE has made great progress in operative techniques and clinical guidance. The increasing use of propofol and target controlled infusion, with the Bispectral Index (BIS), capnography, oxygen saturation monitoring technologies, helped provide induced sleep similar to natural sleep. UA characteristics classifications were created and the association between the collapse patterns and the surgical success rate was found. Recently, DISE has also been performed on individuals without surgical indications, such as intolerance to continuous positive airway pressure (CPAP) therapy, suspected positional OSA and possible intraoral device candidates. A series of position papers has been published, moreover, more physicians have recognized the importance of identifying collapse characteristics through DISE [4–6].

## 2. Concept and terminologies

This procedure was first introduced as sleep nasoendoscopy. Several nomenclatures have been applied, such as video sleep nasoendoscopy, sleep endoscopy^,^ and fibre-optic sleep endoscopy [7–10]. However, the term DISE has been recommended and widely used after the publication of an international consensus paper by De Vito et al. [4], it provided a better description of the use of sedation during the study.

## 3. Indications and contraindications

DISE is a complementary exam in the evaluation of the patient with OSA and should never be used as a substitute for polysomnography (PSG) because it does not provide information about the types of respiratory events and the severity of OSA. The PSG should be accomplished before DISE.

### 3.1. Indications

Indications include (1) patients with OSA and the need for non-CPAP treatment [11]. (2) Patients with previous surgical failure and agreement for OA or surgical intervention assessment [12,13]. (3) Children with residual OSA after adenotonsillectomy [14]. (4) Patients with CPAP failure or intolerance need to identify the reasons of failure or need alternative CPAP pressure titration [15].

### 3.2. Contraindications

Absolute contraindications include American Society of Anaesthesiologist (ASA) type IV: high-risk patients with severe systemic disorders that put the patient’s life at risk, Pregnancy, Previous history of allergy to sleep inducing-drugs. Relative contraindications include morbid obesity and tonsillar hypertrophy grade IV [5].

## 4. Equipment and monitoring

Recommended monitoring during DISE includes oxygen saturation (SaO_2_), electrocardiogram (ECG), and blood pressure (BP). A video-endoscopy system with a flexible nasoendoscope 4 mm in diameter or smaller can be used. Other suggested supplies and equipment include: (i) a standard infusion pump, preferably with target-controlled infusion (TCI). TCI is more effective and safer, allowing better adjustment of the infusion speed [16]; and (ii) a monitoring system for electroencephalogram (EEG)-derived indices - Bi-Spectral Index (BIS) or Cerebral State Index (CSI) [5]. BIS can assist in controlling the level of consciousness and the depth of sedation to mimic natural sleep, with recommended rates of 50–60 [17,18]. Cardiorespiratory polygraphy is suggested for identifying obstructive respiratory events due to hypopneas [19].

## 5. Preparation

Nasal decongestant, topical anaesthesia and anti-secretion drugs are optional preparatory measures to reduce nasal irritation. However, these drugs should be used with caution so as not to interfere with nasal resistance and airflow during physiological sleep, atropine should be avoided [6]. A suction device may be required to drain excess saliva during observation, especially when midazolam is administered as a sedative [5,20].

## 6. Examiners

At least three professionals are required during the exam: one anaesthesiologist monitors the patient, one physician performs the procedure, usually an otorhinolaryngologist, and one physician is present for emergency situations. The endoscopy begins from a complete and stable cycle of snoring or obstruction (hypopnoea or apnoea) with oxygen desaturation and respiration. It is recommended to record two complete cycles for each UA segment, including during manoeuvres, and the number of these cycles should be increased when the administration is performed in bolus [5].

## 7. Sleep-inducing drugs

The isolated use of sleep-inducing drugs is the most common pattern [4]. Fentanyl, remifentanil and ketamine drugs are not recommended to be combined with propofol or midazolam due to increased oxygen desaturation. In 2017, in the European consensus update, midazolam and propofol were recommended as the only sedatives that have a collapsibility value similar to that of natural sleep and do not significantly influence AHI [5]. Dexmedetomidine was once considered the first choice for DISE due to its lower risk of respiratory depression and hypoxia since it was initially used in 2015, except for allowing the appearance of N3 and rapid eye movement sleep [21]. However, compared with midazolam and propofol, dexmedetomidine takes longer to induce sleep and has a protective effect on the airway collapse. When midazolam, propofol, and dexmedetomidine were used separately as sedatives in the same patient, reducing tongue base collapse and hypopharyngeal obstruction was found with dexmedetomidine [22]. The usage of propofol was shown to have similar obstruction sites and patterns to natural sleep, shorter sleep onset latency and higher success rate of sedation observations [23]. Therefore, dexmedetomidine was not recommended as the first-choice sleep inducer agent. However, it had been suggested that propofol acted through some unnatural sleep neural pathways, resulting in changes in cortical processes and sleep architecture, more obvious respiratory depression, significantly lower dilation muscle tone, and lower O_2_ saturation during endoscopic observation. To avoid the effects of propofol overdose on neural pathway and muscle tone, it was recommended to use the TCI system and monitor the process of induced sleep to avoid severe hypoxaemia adverse effects [24,25]. Correspondingly, some surgeons were concerned that higher proportion of tongue falling back after propofol sedation might lead to over-judgement of the need for tongue base surgery, and therefore preferred dexmedetomidine because of its safer hemodynamic stability and less interference with retroglossal collapsibility [26,27]. More randomized prospective studies are needed to resolve the controversy.

Given the modes of medication administration (bolus, pump and TCI, the preferred and recommended option is the TCI [5]. Continuous monitoring and care, avoiding overdose and excessive muscular relaxation are essential for qualifying examinations. Based on the recommendations in the European consensus update in 2017, it is worth highlighting the use of propofol with TCI with an initial dose of 2 to 2.5 mcg/ml, followed by an increase of 0.2 to 0.5 mcg/ml every 2 min. However, an initial target dose of 1.5 mcg/ml can extend the observation range, increase the safety of the exam and obtain enough observation time.

## 8. Positions and manoeuvres

The primary and standard position recommended for DISE is supine, with or without a pillow. When there is a clinical history of snoring or positional OSA, DISE can be started from the lateral position and can then be continued in the supine position. It should be noted that a head rotation might not give the exact same result as a lateral position of the whole body [28]. To evaluate the effectiveness of positional therapy or oral appliance, it is recommended to perform manoeuvres of lateral position or mandibular advancement and vertical opening of the mouth during DISE to achieve direct observation of the therapeutic effect and optimize the patient selection [4,5]. If adjustable OA will be used during DISE to evaluate the efficacy and/or viability of the OA advancement or maxillomandibular advancement surgery, it is recommended to wear the OA before the start of sedation.

## 9. CPAP and DISE

Although CPAP is the first line treatment of OSA, the adherence is approximately 50% in general due to mask discomfort, nasal obstruction, dryness, suffocation, claustrophobia and high positive pressure [29,30].

DISE and CPAP can be performed simultaneously, which allows us to understand and identify unresolved UA collapse under CPAP therapy. It had been found that a higher CPAP pressure (>15 cmH_2_O) was usually required to maintain retrolingual airway patency, widely higher than at the retropalatal level (<10 cmH_2_O) [31]. Moreover, high positive pressure can cause or exacerbate epiglottis collapse [32]. The assessment of CPAP failure should start from not outputting positive air pressure and then gradually transition to output positive air pressure. The mask must be worn on the face when the patient is awake and tested to verify that it is without leakage. The effectiveness of different masks can be evaluated separately during DISE [33]. The evaluation starts from the pressure value obtained by CPAP titration, or the actual value used by the patient. It is suggested to gradually increase 1 cm H_2_O with each apnoea until the airway lumen achieves complete stabilization [34]. However, it is not recommended to apply DISE for titration to replace other well-accepted methods [15].

## 10. Documentation

The documentation of the examination record is recommended; usually, only videos or images of UA are stored. In 2017, Gobbi et al. [19] proposed a polygraph to capture, fuse, display and store images of UA obstructions and cardiorespiratory parameters simultaneously during DISE. In 2018, Dijemeni and Kotech also designed a system that could simultaneously display UA dynamic video and cardiovascular parameters, called DISE Data Fusion [35].

## 11. Classification systems

DISE findings classification systems play important roles in clinical analysis, comparison, and treatment decision-making. It is recommended that a classification system should include at least the following characteristics: obstructive level (and/or structure), degree (severity of airway obstruction) and configuration (direction of collapse) [7]. Thus far, 19 classification systems have been described. However, there is still no standard classification [36].

Two classifications stood out earlier: VOTE (Velum, Oropharynx, Tongue base and Epiglottis) and NOHL (Nose, Oropharynx, Hypopharynx and Larynx) [12,37]. The discussion and comparison between these two systems mainly focussed on the classification of obstructive sites; VOTE was thought to have more comprehensive analysis on the epiglottis and pharynx than NOHL [38]. Later, the universal Drug-Induced Sedation Endoscopy (u-DISE) classification was created to combine the results obtained in the above classifications [39]. It consists of seven sites (nose, soft palate, tonsils, lateral pharyngeal/oropharyngeal wall, tongue base, epiglottis and larynx), compared with four sites of VOTE and NOHL. Although there is no consensus, the VOTE classification is commonly used for data presentation in publications, while NOHL and u-DISE are used less often. The classifications of VOTE, NOHL and u-DISE are shown in Table 1.

**Table 1.** VOTE, NOHL and u-DISE scoring system.

Classification	Obstructive sites	Degree of obstruction (lumen reduction)	Patterns or configuration of collapse			
VOTE	Velum^a^	0, <50%. 1, 50–75%. 2, >75%.	AP	L		C
	Oropharynx^b^			L		
	Tongue base^c^		AP			
	Epiglottis		AP	L		

NOHL	Nose^d^	1, 0–25% 2, 25–50% 3, 50–75% 4, 75–100%				
	Oropharynx^a^		AP	T		C
	Hypopharynx^c^		AP	T		C
	Larynx	Positive, Negative	Supraglottic		Glottic	
	Tonsils^e^	Grade 3, Grade 4				

u-DISE	Nose^d^	0, <50%. 1, 50–75%. 2, >75%.				
	Velum^a^		AP	L		C
	Tonsils^e^			L		
	Lateral pharyngeal wall^b^			L		
	Tongue base^c^		AP	L		C
	Epiglottis		AP	L		
	Larynx		Supraglottic		Glottic	

AP: anterior-posterior, anterior structures moving posteriorly against the posterior pharyngeal wall; L or T: lateral or transversal, lateral pharyngeal structures moving towards to the midline of the lumen; C: concentric, a combination of anterior–posterior plus lateral wall movement.

Details of VOTE, NOHL and u-DISE classifications could be found in the references [12,35,37].

^a^Retropalatal space.

^b^Oropharyngeal lateral wall collapse.

^c^Base of tongue space.

^d^Nasal cavity.

^e^Palatine tonsils.

**Figure 1.**
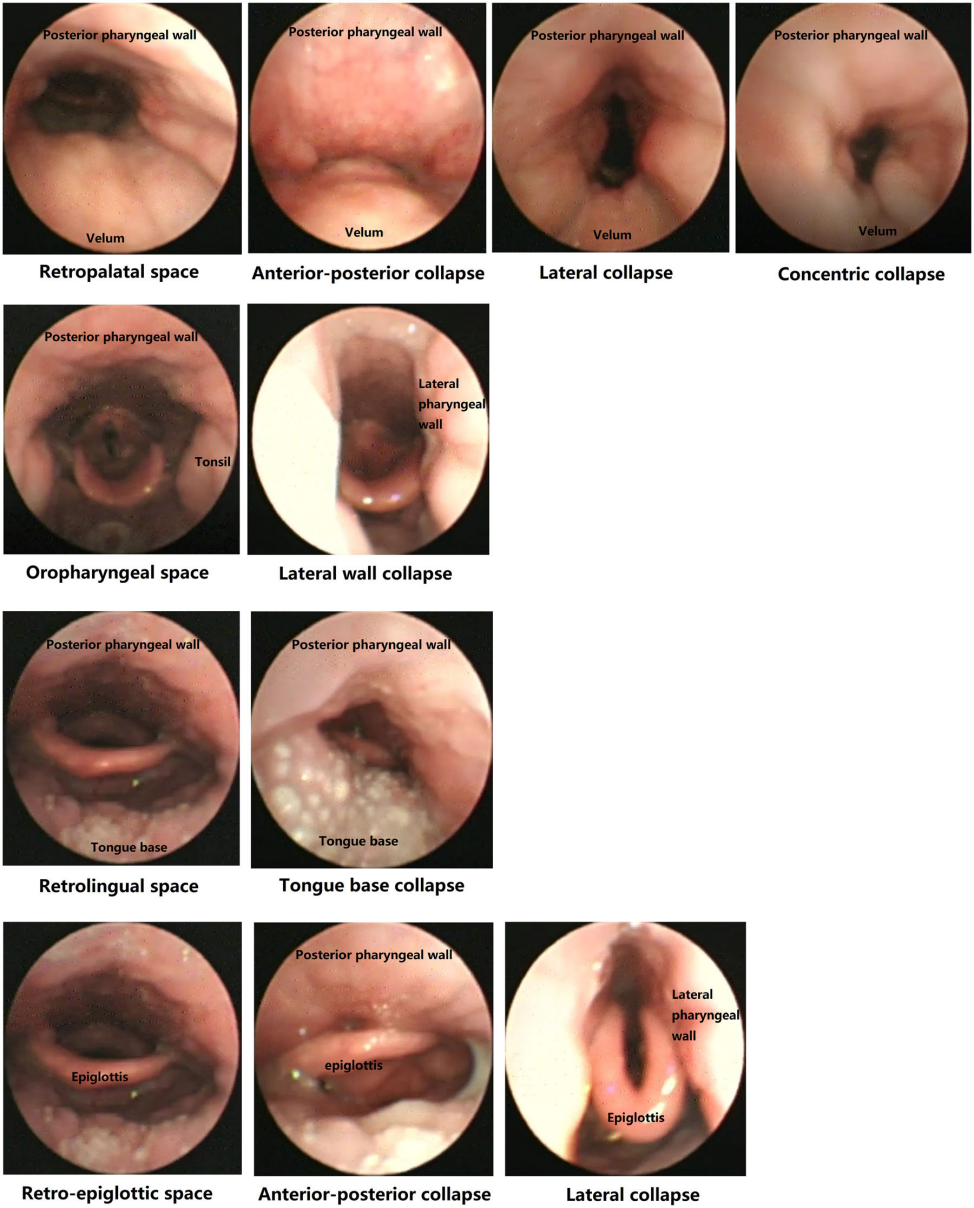
Collapse configurations of different obstructive sites. Airway configurations in velum, lateral oropharyngeal wall, tongue base and epiglottis during normal breathing and inspiratory apnoea were shown in the figure. The leftmost pane showed the normal airway as normal breathing. Inspiratory airway collapse occurred during obstructive apnoea, various configurations were showed on the right, that included anterior-posterior, anterior structures moving posteriorly against the posterior pharyngeal wall; lateral, lateral pharyngeal structures moving towards to the midline of the lumen; concentric, a combination of anterior–posterior plus lateral wall movement. The images were taken from the DISE videos of the authors’ unit to illustrate the different collapse patterns. The DISE was operated by author C.Z, and images were provided by the author C.Z. No patient privacy or informed consent was involved, and no permissions were required for accessing these images.

In relation to obstructive structures in UA, VOTE uses sites (velum, oropharynx, tongue base and epiglottis), others use levels [12,37,39]. VOTE describes 3 degrees of severity: 0, no obstruction (<50%); 1, partial obstruction (50-75%) and 2, complete obstruction (>75%) [12]. NOHL uses a semiquantitative classification: grade 1 (0-25%); grade 2 (25-50%), grade 3 (50-75%); and grade 4 (75-100%) [37]. The configuration of the obstruction is similar in different classifications, including anteroposterior (AP), lateral or transverse (L) and concentric or circular (C). These configurations are shown in Figure 1. The authors believe that the simplicity of the VOTE classification makes the results between observers more consistent, while it contains fewer possible or rare forms of obstruction [12].

## 12. Clinical significances of DISE

Regarding to surgeries, based on the sites, configurations, and severity of UA collapse through DISE observation, it is possible to predict the outcome of UA surgery. Although the evidence supporting DISE prior to surgery was scarce and inconclusive [40–42], some studies demonstrated that the efficacy of OSA surgery could be predicted based on collapse configurations [43,44]. Koutsourelakis et al. [44] found that in a sample of 49 OSA patients who underwent DISE, surgical treatment and postsurgical PSG, the presence of complete circumferential collapse (CCC) of the soft palate and complete anteroposterior collapse of the tongue base was associated with a high failure rate of UA surgery. Chiu et al. and Hsu et al. found that the baseline retropalatal CCC was associated with a higher residual AHI, that was almost twice than non-CCC individuals, after modified uvulopalatopharyngoplasty or advanced palatopharyngoplasty [42,45]. The configurations such as lateral collapse of oropharyngeal wall and AP collapse of epiglottis also had a higher prevalence in patients with failed surgery, 73% and 93%, respectively. In patients with successful surgery, the proportion dropped to 37% and 63%, respectively [46]. In a multicenter cohort study, the association between preoperative DISE and postoperative PSG outcomes was analysed retrospectively in 275 patients undergoing palatal and tongue base surgery. The results showed that complete lateral pharyngeal wall collapse or tongue base collapse reduced surgical success by nearly 50% [47]. Kezirian EJ applied DISE to observe the post-surgical UA collapse features in 33 individuals with uvulopalatopharyngoplasty(UPPP) failure and found that 45% of patients still had complete retropalatal collapse and 79% of patients had complete hypopharyngeal obstruction, confirming that residual palatal and glossal obstruction were important factors affecting the efficacy of UPPP [48]. Based on preoperative DISE assessment, surgeons could identify isolated velopharyngeal obstruction from combined velopharyngeal-hypopharyngeal obstruction, increasing the success rate of modified barbed pharyngoplasty from 60% to 83% [49]. In a study comparing two OSA cohorts with similar demographics and sleep test outcomes, compared with the group without pre-surgical DISE, preoperative DISE not only improved surgical success rate (86% vs 51%), but also decreased the proportion of multilevel surgeries (8% Vs 60%), that actually reduced unnecessary surgical damage and the risk of complications [50]. For patients with retroglossal obstruction proved by DISE, tongue base resection could be performed as part of multilevel surgeries, significantly improved AHI and nocturnal oxygen desaturation [40].

Regarding CPAP therapy, DISE was not recommended for titration, but could be used to identify the causes of CPAP failure. The flexible endoscope could be inserted into the mask through bronchoscopy adapter or from the underside of the mask, and then into the nasal cavity for DISE. While CPAP was turned on, UA dynamic changes under continuous positive pressure could be assessed. It had been found that the positive pressure required to maintain compliance of the lateral pharyngeal wall was lower than the anteroposterior collapse of the tongue base, then was velum or epiglottis [31]. For patients with CPAP failure, it could be found that under continuous positive pressure, persistent epiglottis or tongue base collapse would still occurred, which was more difficult to relieve if accompanied by open-mouth breathing [51,52]. In these cases, a higher positive pressure therapy is necessary for the mobilization of the obstruction, reducing the therapy compliance by the patient. In contrast, the velum and lateral oropharyngeal wall showed better compliance with positive pressure therapy [53]. In these patients, DISE became relevant when it allowed the identification of sites and their obstructive characteristics or patterns of obstruction, defining the pattern most compatible with positive pressure therapy.

Regarding OA therapy (OAT), DISE showed to be a useful tool for screening responders, measuring the distance of mandible advancement, and improving OAT efficiency. In a clinical observative study, 35 patients with incomplete response to OAT (residual AHI >15 or had obvious subjective symptoms) were performed DISE without and with OA in place, respectively. The proportion of velum, lateral pharyngeal wall, tongue base and epiglottis collapse decreased from 91%, 43%, 31%, and 31% at baseline to 43%, 11%, 9%, and 20% after wearing OA. The velum and epiglottis collapse had less improvement and more residual, thought to be associated with OAT failure [54]. Another study collected 72 OSA patients, 33 had good response with OAT (>50% reduction in AHI), 22 had no response, 17 became worse (>10% increase in AHI). It was found that the baseline tongue base collapse had 3.7 increased odds of responding to OAT, but the retropalatal CCC and complete lateral pharyngeal wall collapse (LPW) resulted in 5.3 and 6.6 decreased odds [55]. The simulation of mandibular advancement could be performed during DISE to assess the actual effect of OAT. One way was to wear a custom-made simulation bite with maximal comfortable protrusion of the mandible during DISE. In cases where the OA device did not modify the AHI, DISE plays a relevant role in identifying the advancement of the tongue and epiglottis collapse. If persistent collapse of the epiglottis and lateral pharyngeal wall was observed, it usually indicated more residual AHI [56,57]. The other method was to use manually controlled titratable mandibular positioner, called Selector Avance Mandibular (SAM) device. The advancement distance could be gradually increased during DISE until the UA collapse were relieved, and then the actual range of OAT could be predicted. If residual or worse collapse remained under DISE + SAM, OA would not be recommended [58]. Apart from the SAM device for OAT titration, another device (MATRx) could also be used to perform OAT titrations during DISE [59]. Jaw thrust was a simple way to mimic the OAT. This manoeuvre was performed with hands behind the mandible angle and jaw pushed anteriorly [11]. A prospective, cohort study was designed to evaluate this manoeuvre. The effects of jaw thrust and actual OA in the same patient was compared during DISE. The airway collapse and snoring were significantly improved in 13 patients after jaw thrust, and 4 patients had residual soft palate flutter. After wearing OA, airway obstruction and snoring disappeared completely in 3 cases, was improved in 9 cases, and persisted in 5 cases [60]. Patients with significant retropalatal and retroglossal airway dilation after jaw thrust would have a 75%-79% success rate (AHI < 15) with actual OAT. Without jaw thrust + DISE, the success rate was only 50% [61,62]. Jaw thrust appeared to be in good agreement with actual OA and was helpful in screening the OAT responders. However, compared with OA in place, the jaw thrust manoeuvre only increased the anteriorly advancement but lacked vertical movement of mandible, resulting in greater dilation in tongue base region and less in the retropalatal region [63,64]. Therefore, it was necessary to emphasize the difference between jaw thrust and actual OAT when the simulation results were analysed. Overall, pre-OAT DISE was a good screening and predicting tool that could significantly improve AHI and quality of life compared to non-DISE management [65].

Regarding maxillomandibular advancement (MMA), DISE provided new insights into skeletal surgery for OSA. As mentioned above, baseline retropalatal CCC during DISE was considered a predictor of non-response or failure in soft tissue surgery or OAT. However, for MMA, CCC was not a contraindication. In a prospective case series study, DISE outcomes before and after MMA were compared. Pre-MMA retropalatal CCC were all completely alleviated, and the AHI improvement in individuals with CCC was equal to those without CCC [66]. Based on the DISE findings, surgeons also found that the oropharyngeal lateral wall collapse usually had the most improvement after successful MMA, it was the increase in lateral wall tension but not only the increase in anterior-posterior space in pharyngeal airway was associated with success rate. Therefore, a baseline lateral wall collapse or retropalatal CCC could be considered as indicators of MMA [67]. On the other hand, the epiglottis collapse was found to be a negative predictor for MMA. In a retrospective cohort study, the pre-MMA DISE outcomes were compared between 39 responders and 25 nonresponders, the significant negative factor to affect the MMA efficiency was the complete AP epiglottic collapse, that would cause 0.239 lower odds for surgical success [68].

Regarding trans-oral robotic surgery (TORS), DISE indicated that lateral velopharyngeal collapse was a potential Influencing factor for TORS tongue base reduction and/or multilevel surgery. Patients without lateral wall collapse had higher response rate (66.7%). Prospective studies with larger samples were expected to fully evaluate the predictive mode of DISE for TORS [69].

Regarding hypoglossal nerve stimulation (HNS), DISE was considered a routine preoperative screening assessment. Moderate to severe OSA patients without CCC at the level of the soft palate were found to have satisfactory effects after long term follow-up. CCC had been widely accepted as a contraindication for neurostimulator implanting [70]. However, it had recently been pointed out that CCC might not be a contraindication to bilateral HNS devices, but more observations were still needed [71]. Besides CCC, other collapse patterns were also shown to correlate with HNS outcomes. Tongue base collapse had been shown to be associated with higher response rate, but the lateral wall collapse was associated with lower response rate [72]. OA could also be applied during DISE to predict the efficiency of HNS, and individuals sensitive to OA were found to response well to HNS [73].

## 13. Discussion

The pathophysiology of OSA is attributed to various factors, such as anatomy, central nervous system control, respiratory feedback and myopathy, ultimately manifesting as UA collapse during sleep. Although CPAP is the most common treatment, alternative therapies are also required for patients with low adherence and intolerance. Therefore, understanding the changes in UA compliance during sleep is very helpful for physicians to identify causes and then propose targeted treatment plans. DISE provides a unique viewing mode to directly examine continuous dynamic changes in the airway lumen during sleep. In the past 20 years, research on targeted therapy for DISE subtypes has increased year by year, and a series of position papers have been proposed. Through DISE, the phenotype of UA obstruction has been further refined according to various sites and collapse configurations, going beyond conventional definitions such as "pharyngeal cavity narrowing" or "tongue hypertrophy". Surgeons have become accustomed to using DISE to assess surgical indications and improve outcomes. At the same time, the complex regulatory mechanism of UA collapse has also been recognized, and future treatments are far from simply correcting anatomical deformities. The most interesting advances are combining DISE with conservative therapies (positional therapy, CPAP, OA) and novel therapies (bone reconstruction, robotic-assisting surgery, neurostimulator implantation) to improve accuracy and effectiveness.

DISE concurrent with wearing CPAP and OA can help physicians understand how UA obstruction is actually improving; whether the degree of improvement can reach expectation; and if it fails, what are reasons and what would be subsequent solutions. The manoeuvres of head rotation or protrusion of mandibular during DISE can predict the effectiveness of postural therapy or mandibular advancement therapy. Several innovative treatments have in turn increase acceptance of DISE among sleep medicine physicians, for example, the success of hypoglossal nerve stimulator implantation clearly demonstrating the necessity of DISE in screening candidates to exclude velopharyngeal complete concentric collapse. The complete lateral pharyngeal collapse can be restored after maxillomandibular advancement.

However, there were some limitations of DISE. No studies have been able to identify the presence of REM sleep during DISE with midazolam, propofol and/or dexmedetomidine. Heo et al. proved that obstructive patterns may change if the observation duration is too long [74]. However, the obstruction characteristics will not be fully observed in a shorter period. As demonstrated by the study, a 15-min duration seems to be more appropriate [74]. The correlation between obstruction sites and PSG results, such as AHI and oxygen desaturation index (ODI), is quite uncertain. DISE study has shown that multiple-site obstruction was correlated with OSA severity [75]. However, another study demonstrated that the number of sites with complete obstruction was not associated with AHI and ODI [76]. Supine position is also a limiting factor because it does not reflect the actual effect of decubitus on UA collapse during all-night natural sleep [77]. The evaluation of the exam is subjective and depends on the training or experience of the observer, the variation of DISE results between different observers was noted [78]. Moreover, the pharmacological difference of medications can also affect the results, leading to over- or underestimating UA collapse due to the selection of sedative drugs during DISE [79].

Currently, DISE is mainly performed in the operating room and requires sedation by an anaesthesiologist, post-anaesthesia monitoring by a nursing team, and endoscopic operation by an experienced otolaryngologist, which allows for more accurate and reliable judgement of obstructive sites, patterns, and degree [80]. However, these also make DISE more medical resource intensive, resulting in higher costs, and difficult to be performed as a routine OSA examination in most hospitals. DISE was usually performed only in patients with surgical indication. To simplify the process, a few institutions plan to perform DISE in the department of otolaryngology in an outpatient setting. However, reliable sedation requires more than sedative drugs and TCI, the anaesthesiologist plays an important role in ensuring the safety and avoiding complications such as respiratory depression. In the author’s opinion, coordinating the work schedules of doctors in different departments is not easy. At the same time, a separate operating room and recovery room are also required with adequate monitoring and recovery after anaesthesia. Therefore, few patients can have access to DISE in an outpatient setting. Meanwhile, the lack of the multidisciplinary participation, such as sleep medicine and respiratory physicians, maxillofacial surgeons, orthodontists, etc. limits the promotion of DISE. In addition, there are contradictions about the guidance of DISE on treatment. Some studies confirm that DISE-based decision-making improves response rates, while others not. This maybe attributes to requirements discrepancy, such as sedative drugs, duration of operation, classification of records, and treatment options also depend on personal experience, that makes the comparison between studies difficult. Therefore, establishing unified standard, improving operability, increasing multidisciplinary participation will be beneficial to the development of DISE.

There are clinical parameters such as sex, body mass index (BMI), race, drug addicts, type of bite and face, craniofacial deformities, neuromuscular and degenerative diseases, use of certain medications, severity of apnoea, percentage of desaturations and findings of endoscopic examinations with the patient in wakefulness that have been identified in the studies to date to correlate with DISE findings. For example, some findings in nasopharyngolaryngoscopy could predict DISE results, especially the fall of the epiglottis on forced inspiration. Authors showed that patients with CCC of the soft palate were significantly more likely to have higher AHI and BMI, while anteroposterior velar collapse was significantly associated with a lower BMI. Moreover, the AHI was significantly higher in patients with complete anterior-posterior collapse of the tongue. Lower AHI was associated with a higher probability of a partial concentric collapse [6,55]. These parameters could be analysed in isolated form or associated corroborate for alterations in superior and/or inferior levels of the airway that allow us an individualized therapeutic approach and with higher success rates.

Generally, compared to other dynamic examinations such as intraluminal pressure monitoring, cine CT or MRI, DISE has advantages in the balance of accuracy, operability, cost, and efficiency, it will remain the focus of OSA research. More prospective, randomized controlled studies are needed to guide the clinical practice. In the future, synchronous EMG and EEG monitoring during DISE will provide insight into the myopathy and neuropathy of airway collapse. The invention of tiny, less irritating instrument is expected to enable endoscopy in true natural sleep.

## 14. Conclusions

Despite the limitations of DISE itself, it is undeniable that it has become the most common and most widely performed OSA evaluation method. DISE not only provides clinicians with valuable information about the sites, pattern and degree of obstruction but also provides profound insights into the UA collapse mechanism during sleep. Considering that DISE is closely involved with innovative therapies such as hypoglossal nerve stimulation and TORS, it is likely that DISE will play a more important role in guiding clinical practice in the future.

## Author contributions

AV and CZ contributed to the concept and design of the study. DE carried out the materials analyses. AV and DE contributed to the manuscript editing and writing. CZ contributed to the manuscript revising. All authors contributed to the manuscript approving.

## Data Availability

Data sharing is not applicable to this article as no new data were created or analysed in this study.
